# Evidence-Based Lifestyle Guidelines and Self-Management Strategies Utilized by Women with Polycystic Ovary Syndrome

**DOI:** 10.3390/nu15030589

**Published:** 2023-01-22

**Authors:** Stephanie Cowan, Angela Grassi, Lynn Monahan Couch, Yvonne Jeanes, Siew Lim, Stephanie Pirotta, Jeff Harris, Caroline McGirr, Lisa Moran

**Affiliations:** 1 Monash Centre for Health Research and Implementation, School of Public Health and Preventive Medicine, Monash University, Clayton, VIC 3168, Australia; 2 Nutrition Department, West Chester University of Pennsylvania, West Chester, PA 19383, USA; 3 School of Life and Health Sciences, Department of Life Sciences, University of Roehampton, London SW15 5PH, UK; 4 Eastern Health Clinical School, Monash University, Box Hill, VIC 3128, Australia; 5 Health and Social Care Unit, School of Public Health and Preventive Medicine, Monash University, Melbourne, VIC 3004, Australia

**Keywords:** polycystic ovary syndrome, physical activity, diet, self-management, behavior change

## Abstract

Polycystic ovary syndrome (PCOS) is a complex endocrine disorder, affecting 13% of reproductive-aged women. While lifestyle management is the first-line treatment for improving complications, women experience challenges with implementation. This cross-sectional study aims to identify the types and sources of dietary and physical activity (PA) interventions implemented by women with PCOS and understand how they use self-management strategies to support lifestyle change. An online questionnaire was disseminated via a consumer-based PCOS website (May 2015–2016). Women (n = 1167) were aged 18–45 years and primarily born within the United States (70%). A quarter or less of women (diet 25%, PA 14%) sought lifestyle advice from health professionals (medical clinicians or dietitians) compared to over half (diet 59%, PA 67%) using alternative sources, namely from online platforms. While only 33% and 16% of women reported following formal dietary or PA guidelines, respectively, 57% had implemented a ‘special diet’ to manage their condition, many of which were inconsistent with evidence-based practice in PCOS. Participants also displayed a low level of engagement with important self-management behaviors, including goal setting and positive self-talk. These findings suggest that online information may promote inaccurate and ineffective lifestyle advice and emphasize the need to increase engagement with qualified health professionals.

## 1. Introduction

Polycystic ovary syndrome (PCOS) is a complex and common endocrine disorder, affecting up to 13% of reproductive-aged women [1,2]. Features of PCOS include reproductive (infertility and pregnancy complications) [3], metabolic (metabolic syndrome, type 2 diabetes, and cardiovascular disease) [4], and psychological (depression, anxiety, disordered eating, and poor quality of life) complications and morbidity [5,6]. Insulin resistance (IR) is an intrinsic pathophysiological feature in the etiology of PCOS that is mechanistically distinct from IR associated with obesity, affecting up to 75% of lean women with PCOS. However, excess weight also exacerbates the presentation of PCOS likely through the contribution of extrinsic IR [7]. This is concerning given that women with PCOS have a higher prevalence of overweight and obesity [8,9]. There are many proposed mechanisms that predispose women with PCOS to weight gain [10], including the effects of hyperinsulinemia on ovarian androgen production, with hyperandrogenism favoring abdominal fat disposition [11]. While the relationship between IR, hyperandrogenism, and weight is complex and still not fully understood, it is well established that lifestyle management is a first-line treatment in PCOS [12].

The 2018 PCOS Evidence Based Clinical Guidelines recommend lifestyle management for improving reproductive, metabolic, and psychological complications [13]. Lifestyle is a construct that could consider a range of traditional, complementary, and integrative medicine (TCIM) therapies such as psychological and sleep interventions, acupuncture, and supplement use [14]. However, with regards to the 2018 PCOS Evidence Based Guidelines, lifestyle is currently defined as those designed to improve dietary intake or physical activity (PA) [15,16,17] through appropriate behavioral support (e.g., goal setting, monitoring, use of reinforcements) [18,19,20]. While it is important to recognize the broader concepts of lifestyle, this study uses the traditional definition outlined in current guidelines.

Although effective lifestyle management is a key component of PCOS care, evidence of higher longitudinal weight gain in community populations [8] and high attrition rates in clinical weight management interventions [19] suggests that women with PCOS experience challenges with dietary and PA management. This indicates a clear need to better understand how women with PCOS engage with evidence-based dietary and PA interventions. Their exposure to and utilization of information that promotes effective and accurate lifestyle behaviors needs to be better characterized. An international study in 1385 women with PCOS reported that very few women (12%) were satisfied with the information received about lifestyle interventions from their primary health care providers [21]. Emerging research also suggests women with PCOS are resorting to the internet and social media as their primary source of lifestyle information, rather than recognized health professionals [22,23]. As women increasingly turn to online platforms for health education, they are tasked with the job of filtering the accuracy and quality of the enormous quantities of information available online [24]. This exposes them to potentially ineffective and harmful advice [22,23] that may have detrimental impacts on the successful implementation of lifestyle change.

Traditionally, nutrition information was disseminated almost solely by qualified health experts, trained to utilize counselling approaches that employ a range of cognitive (e.g., positive-self talk and goal setting) and behavioral (e.g., having healthy foods easily available) self-management strategies [25] to improve lifestyle outcomes [26,27,28]. Self-management reflects an individual’s responsibility for the daily conduct of health behaviors, helping them to mitigate their own condition through the promotion of self-efficacy (an individual’s belief that they themselves can execute behaviors) [29,30]. Women are particularly vulnerable to psychological factors that can reduce self-efficacy, such as poor self-esteem and self-doubt, which detrimentally affects their ability to implement lifestyle change [31,32]. In order to avoid disease-related complications, regulate symptoms, and reduce the severity of disease, self-management strategies that counter negative thoughts and improve support from family/friends should be targeted in PCOS [20]. However, previous research reported that only a limited number of PCOS websites provided advice on self-management strategies [33]. These findings suggest that online PCOS lifestyle recommendations may not only indorse misinformation but may also fail to promote self-management strategies required to achieve sustainable lifestyle change.

To date, there is a paucity of recent studies that examine the information sources used by women with PCOS when implementing lifestyle modification [22,23]. Even fewer studies have characterized the types of self-management strategies employed to optimize behavioral change in this population [34]. This study aimed to: (i) identify the types and sources of dietary and PA interventions utilized by women with PCOS and (ii) characterize the behavioral and cognitive self-management strategies utilized by women with PCOS when implementing dietary or PA interventions.

## 2. Materials and Methods

### 2.1. Sample Population

In this online cross-sectional study, women with PCOS were recruited (May 2015–May 2016) through the PCOS Nutrition Centre (a consumer-based website) and at two PCOS symposiums (Atlanta and Philadelphia) hosted by the largest non-profit PCOS organization, PCOS Challenge: The National Polycystic Ovary Syndrome Association.

Women were eligible to participate if they were aged 18–45 years, were not pregnant or breastfeeding for up to six months prior to completing the survey, and self-reported a diagnosis of PCOS. This study was conducted according to the guidelines laid down in the Declaration of Helsinki and all procedures involving human subjects were approved by the Institutional Review Board of West Chester University (ID 20151020). Written informed consent was obtained from all participants prior to initiating the survey.

### 2.2. Data Collection

The survey consisted of 97 questions, and the data analyzed in this study were taken from three separate sections: demographics; knowledge and sources of diet and PA information; and self-management strategies for PCOS.

#### 2.2.1. Demographics

Demographic data included self-reported age, race, country of birth, medical history, and anthropometry. Medical history included PCOS diagnosis, symptoms/features of PCOS, medications, and co-morbidities. BMI categories were calculated according to the World Health Organization (WHO) criteria [underweight (<18.5 kg/m^2^), healthy weight (18.5–24.9 kg/m^2^), overweight (25–29.9 kg/m^2^), or obese (≥30.0 kg/m^2^)]. Weight changes were assessed by asking participants if their weight had increased or decreased by more than 5 pounds (2.3 kg) in the past 3 months, of which women answered yes or no.

#### 2.2.2. Knowledge and Sources of Diet and Physical Activity Information

The questions used to assess knowledge and sources of diet and PA information are presented in the Supplementary Material (Appendix A). The questions were developed by the researchers and tested for usability and face validity. Briefly, the questions were piloted on clinicians, researchers, and women with PCOS. Feedback pertaining to their interpretation of the questions was then incorporated into the final questionnaire, which was retested prior to its use in this study.

Participants were asked questions relating to: (i) their awareness of any national and international lifestyle guidelines for PCOS management; (ii) whether they followed any formal diet or PA guidelines to manage their PCOS (e.g., Dietary and Physical Activity Guidelines for Americans [35,36], Dietary Approach to Stop Hypertension (DASH) [37], information provided by the American Diabetes Association [38], and the Physical Activity Guidelines for Americans [36]); (iii) whether they followed any special diets to help manage their PCOS (e.g., low carbohydrate, vegetarian, or gluten free diets); (iv) whether they engaged in regular physical activity; and (v) where they sourced information for the dietary and PA plans they followed (e.g., from health professionals including doctors and registered dietitian nutritionist (RDN) or from alternative sources such as websites and social media).

Most questions were provided in a close-ended multiple-choice format, where participants were asked to choose from a distinct set of pre-defined responses. However, some questions also contained open-ended options that allowed participants to provide a written response. Specifically, for questions relating to the use of formal dietary guidelines and sources of dietary and PA advice, if none of the pre-defined answers were appropriate, participants were given the option to select ‘other’ and provide a written response. For participants who reported engaging in regular PA, they were also given the opportunity to provide a written response outlining the type and/or frequency of PA undertaken.

#### 2.2.3. Self-Management Questionnaire

Diet and PA self-management strategies were assessed using the diet and PA self-management scale derived from the validated scale by Saelens et al. [30]. The questionnaire aims to understand the types and frequencies of self-management strategies used to best manage personal behavior change. This comprised of 16 questions for diet and 12 questions for PA, with each being categorized according to behavior or cognitive strategies. Scoring of each strategy was completed using a Likert scale with a range of 1 (never), 2 (occasionally), 3 (often), 4 (very often), and 5 (always). Higher scores therefore indicated more frequent use of diet and PA self-management strategies.

### 2.3. Data Analysis

All quantitative analyses were completed using the Statistical Package for Social Sciences (SPSS), version 22.0 (IBM, Armonk, NY, USA). Normality of data were assessed visually using histograms. Descriptive statistics for categorical data were reported using frequencies and continuous data (e.g., age and BMI) were reported using means and standard deviations (all continuous data were parametrically distributed). For the self-management questionnaire, mean scores were calculated for each strategy. Overall diet and PA self-management scores were measured as the mean of all 16 diet and 12 PA strategy Likert scales.

Qualitative analyses of open-ended questions were undertaken using conventional content analysis [39]. There were 120 written responses which were independently coded by two researchers (SC and CM) using an inductive approach. Codes were then reduced to categories, providing a clearer understanding of the breadth of responses relating to the types of diet and PA plans followed and the sources of lifestyle information utilized.

## 3. Results

A total of 1627 adult women with self-reported PCOS were recruited and 1167 women (72%) who completed 75% of the questionnaire were included in the final analysis.

### 3.1. Demographics

Demographics and medical history are reported in Table 1 and Table 2. The mean ± SD age of the participants was 32 ± 7 years. They were mostly white (78%) and born in the United States (70%). Body weight and BMI were 206.0 ± 56.4 lb. (93.6 kg) and 34.3 ± 8.9 kg/m^2^, respectively. Most participants were overweight (18%) or obese (65%) and had experienced a weight change of more than 5 lb. in the last 3 months (71%).

Women reported that their PCOS diagnosis was most commonly confirmed by a gynecologist (55%), followed by a general practitioner (21%) and endocrinologist (19%). Menstrual irregularities occurred in 86% of women, with 62% taking medication(s) to regulate their menstrual cycle or promote ovulation (31% using oral contraceptives and 38% metformin). Excess body hair was reported by 81% of participants and acne by 56%. Lastly, several co-morbidities were present, with the most commonly reported conditions including infertility (38%), hypercholesterolemia (15%), and hypertension (15%).

### 3.2. Knowledge and Sources of Diet and Physical Activity Information

Knowledge of formal diet and PA guidelines and special diets followed by participants are reported in Table 3. Almost all (93%) of the respondents were not aware of any specific PCOS national or international lifestyle guidelines. While 33% of women reported having followed formal dietary guidelines, the majority (58%) of these women did not select the pre-defined evidence-based diets listed, instead reporting that they followed ‘other’ guidelines. Content coding of written responses revealed that these ‘other’ guidelines included diets where women had completed their own research and were following recommendations with limited evidence in PCOS management, such as paleo or ketogenic diets. Similarly, over half of women (57%) reported following a ‘special diet’ to help manage their PCOS. Although some of these ’special diets’ were consistent with recommended evidence-based practice for PCOS [13] (such as low glycemic index (37%) and low-energy (20%) diets), many did not reflect recommendations utilized by health professionals in PCOS management (including low-carbohydrate (63%), gluten-free (32%), and dairy-free (30%) diets).

Only 13% of the respondents were aware of the 2008 Physical Activity Guidelines for Americans, and 16% reported following formal PA guidelines to manage their PCOS symptoms. Despite the lack of knowledge and utilization of formal PA guidelines, over half (52%) reported engaging in regular exercise. A combination of strength and cardio training was the most popular type of exercise (59%), followed by a combination of different cardio exercises (16%) and walking (14%).

**Figure 1:**
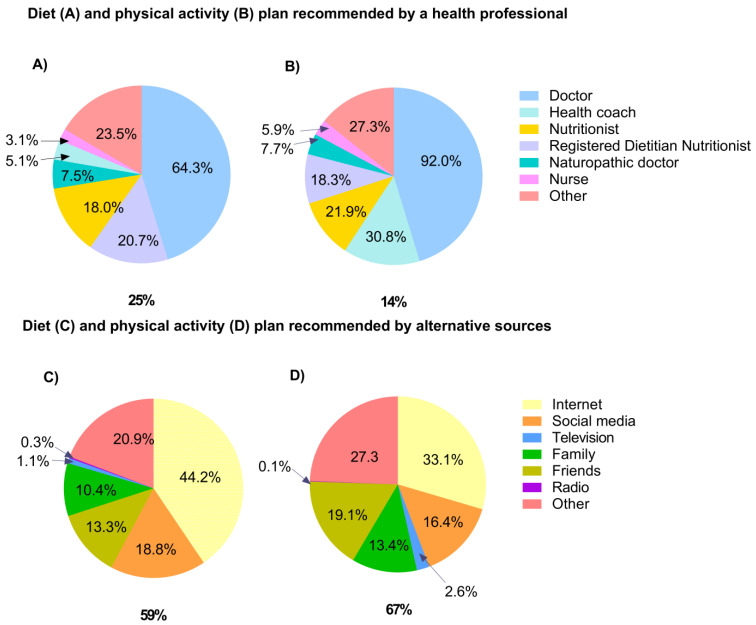
Diet and physical activity plans recommended by qualified health professionals (**A**,**B**). Diet and physical activity plans recommended by alternative information sources (**C**,**D**). Percentages beneath pie charts are expressed as a proportion of the total study sample (n = 1167). All other percentages (within pie charts) are expressed as a proportion of their corresponding category (e.g., of the 14% of women who sourced physical activity recommendations from a health professional, 92% of these women received this information from their doctors). Percentages do not add up to 100% where survey respondents could provide ≥1 answer or where there were missing values.

Sources of dietary and PA recommendations are reported in Figure 1. Dietary plans were recommended by a health professional for 25% of women, with the majority sourcing their information through physicians (64%) and only 24% through an RDN. For the 24% of women who reported sourcing their information from ‘other’ health professionals, content coding of written responses highlighted the scope of health professionals providing dietary advice, including endocrinologists, acupuncturists, and personal trainers (presented in the Supplementary Material, Appendix B, Table A1). Of those sourcing dietary advice through alternative avenues (59%), almost half (44%) accessed dietary recommendations from the internet and 19% specifically used social media websites.

A total of 14% of participants received PA advice from a health professional, with physicians (92%) and health coaches (31%) being the most popular sources of information. Over two-thirds of women (67%) sourced their PA plan from alternative avenues, with half of these women (50%) using the internet and social media. For the 27% of women who reported obtaining their exercise plans from ‘other’ alternative avenues, content coding of written responses revealed 73% were using their own form of research (Supplementary Material, Appendix B, Table A1).

### 3.3. Dietary and Physical Activity Self-Management Strategies

The self-management strategies utilized by participants when implementing diet and PA interventions are reported in Table 4. The overall self-management scores for diet and PA behavioral change were similar (2.99 ± 1.22 and 2.96 ± 1.27, respectively).

When implementing dietary behavioral change, the least frequently utilized strategies across both cognitive and behavioral domains included seeking information from a health professional (2.09 ± 1.19), tracking food intake (2.62 ± 1.23), and using positive self-talk (2.79 ± 1.28). Conversely, the most frequently utilized strategies included reading labels when choosing foods (3.50 ± 1.23), making plans to change diet/drinking habits (3.39 ± 1.10), and looking for information about nutrition and healthy eating (e.g., online, books, magazines, etc.) (3.30 ± 1.31).

When implementing PA behavioral change, women struggled to make back-up plans (2.10 ± 1.20), seek support from friends and family (2.40 ± 1.30), and get back on track after failing to achieve their goals (2.54 ± 1.23). However, participants were more consistent at knowing when to do more activity (3.64 ± 1.14), thinking about the benefits of being active (3.64 ± 1.10), and keeping track of how much PA is undertaken each week (3.39 ± 1.24).

## 4. Discussion

We report here novel findings from a large community-based sample of women with PCOS that the majority of women do not follow formal diet or PA guidelines for PCOS management, use the internet as their primary source of lifestyle information, and display a low level of engagement with self-management behaviors required for optimal PCOS care.

Over two thirds of participants were not following formal evidence-based dietary or PA guidelines to manage their PCOS. As best practice in PCOS care promotes the use of national lifestyle guidelines [35,36], lack of engagement with qualified health professionals and poor uptake of evidence-based recommendations may reflect the general publics’ waning trust and growing disinterest of government-initiated health messages [11,40,41,42]. This growing skepticism of government recommendations may be heightened in women with PCOS, who consider Dietary Guidelines for Americans [22,43,44] and dietary information provided by health professionals [21,22,45,46] to be too broad or inadequate at managing their unique needs. Furthermore, a large international study including 1385 women with PCOS reported that only 12% were satisfied with the lifestyle advice they received through their healthcare provider at the time of diagnosis [21]. The one-size-fits-all approach promoted by generic lifestyle guidelines may be less able to satisfy the preferences of women with PCOS who prefer a more personalized and specific disease management plan [44,47,48].

More than half of participants in this study used the internet and social media as their primary source of diet and PA information. The increased reliance on online platforms for health advice is not a phenomenon unique to PCOS. Previous research has reported that consumers, in particular women, are twice as likely to access nutrition information online than through nutrition professionals [49] and expressed that the online advice was more detailed, specific, and interesting than advice provided by dietitians [49]. However, as the general public lacks the training and skills required to appropriately evaluate health content, they may struggle to distinguish between biased sources and evidence-based information [50]. This is especially true for online health content, where unlimited and autonomous access to unregulated online platforms exposes the public to a large amount of inaccurate information [24,51]. Previous research by Chiu et al. [52] reported only a limited number of PCOS websites provide accurate and reliable lifestyle management information in accordance with evidence-based guidelines. This paucity may contribute to misconceptions surrounding a healthy lifestyle. Previous research reported women with PCOS follow a range of unhealthy and potentially ineffective lifestyle habits to manage their condition [53], which is supported by our findings that participants followed diets promoting the exclusion of core food groups, including paleo, ketogenic, and elimination diets. While some of these diets (e.g., low FODMAP and dairy free) may be indicated in certain medical conditions (e.g., irritable bowel syndrome or food allergy management), overall, there is limited evidence available to support their efficacy in PCOS management [54]. Furthermore, for the almost half (42%) of women who selected ‘other’ when reporting they followed formal dietary guidelines to help manage their PCOS, many of these women referred to dietary advice that was inconsistent with established recommendations (e.g., following paleo and ketogenic diets). This further suggests that women may be confused as to what dietary strategies are considered best practice in PCOS management.

Information obtained through online websites and social media sites may also fail to incorporate important self-management techniques required for sustainable behavioral change [55,56,57,58]. Previous research has reported only 53% of websites providing lifestyle advice for PCOS management recommended supportive measures for behavioral modification, including goal setting, self-monitoring, and social support [33]. This indicates almost half of the lifestyle recommendations available on PCOS websites do not promote self-efficacy [29] to implement and sustain behavioral change [59,60]. By contrast, health professionals, including dietitians, physiotherapists, and exercise physiologists, are ideally trained to implement theory-based behavioral change strategies that acknowledge and foster responsibility for self-care [59,61,62]. In the present study, there were a number of self-management strategies that were only occasionally utilized by participants, including seeking support from health professionals, seeking support from friends and family, and positive self-talk. These all relate to behavioral strategies outlined within social cognitive theory [63] and self-regulation models of behavior change [64], which may aid in improving the psychological features (e.g., poor body image and self-esteem) thought to detrimentally impact women’s self-efficacy [31,32].

## 5. Strengths and Limitations

We acknowledge this study relied on participants’ self-report of a PCOS diagnosis with no specific inclusion or exclusion criteria. However, with 66% of participants reporting irregular or absent menstrual cycles, and previous research showing a positive correlation between self-reported irregular menstrual cycles and a formal PCOS diagnosis [8], this would limit misclassification of PCOS. This study was also limited by the possibility of self-selection bias, particularly by those with internet access and interest in study participation. Research was conducted predominantly in women residing within the United States who were fluent in English. The population was not racially or ethnically diverse and findings may therefore not be generalizable to real-world community settings. We also recognize that future exploration regarding the uptake and utilization of lifestyle interventions should include a broader range of lifestyle therapies such as sleep and TCIM, especially given growing consumer interest in more holistic approaches to care [14].

This study also has several strengths. This is one of very few studies to characterize the sources of evidence-based and non-evidence-based lifestyle information and self-management strategies utilized by women with PCOS when implementing behavioral change. We were able to reach a large sample size of women residing within the community. As the survey was voluntary and anonymous, the results are likely to reflect the authentic views of women with PCOS in the community. A validated questionnaire was used to assess self-management strategies.

## 6. Conclusions

Our research has demonstrated that healthcare professionals are not the primary source of lifestyle information for women with PCOS, with the internet and social media more commonly utilized for diet and PA advice. This may expose women with PCOS to misinformation that can contribute to unhealthy and/or ineffective lifestyle behaviors. It also may diminish their use of important self-management strategies, having detrimental effects on their self-efficacy and ability to sustain behavioral change over the longer-term.

## 7. Future Recommendations

As gynecologists, endocrinologists, and general practitioners are often the first line of contact following a PCOS diagnosis, they are best placed to improve the uptake of evidence-based lifestyle recommendations and support behavioral change. However, research suggests doctors have limited time in consultations and may lack confidence in discussing lifestyle management [65]. It is therefore crucial to improve the use of referral strategies to allied health professionals (including dietitians, exercise physiologists, and psychologists) who can support the adoption of intensive and complex lifestyle interventions [13]. PCOS is a heterogenous group and future research should also investigate the efficacy of lifestyle and medical management across clinical phenotypes to improve treatment responsiveness and tailoring [66,67,68]. There is also a clear need for health professionals to diversify their communication and increase their engagement with online PCOS-focused lifestyle content. Working alongside women to improve the acceptability of online evidence-based resources may help to increase patient access to reliable and credible online education and online forums for social support [69].

## Figures and Tables

**Table 1:** Demographic characteristics of women with polycystic ovary syndrome.

Variable	Value ^a^
**Weight (lb.)**	206.0 ± 56.4
**BMI (kg/m^2^)**	34.3 ± 8.9
**Weight category**	
Underweight	0.02 (13)
Normal	14.7 (169)
Overweight	18.2 (209)
Obesity	65.0 (747)
Class I	33.6 (251)
Class II	28.9 (216)
Class III	37.5 (280)
**Experienced weight change > 5 lb. in last 3 months**	71.0 (892)
Weight loss	30.5 (356)
Weight gain	42.7 (498)
**Race**	
White	78.4 (905)
Black	6.7 (77)
Asian	3.5 (40)
Other	11.4 (101)
**Country of birth**	
United States	70.0 (817)
Australia	4.7 (55)
United Kingdom	4.3 (50)
Canada	3.3 (38)
Other	17.7 (207)

^a^ Data were reported using mean ± SD or % (N). Percentages are expressed as a proportion of the total study sample (n = 1167). Percentages do not add up to 100% where survey respondents could provide ≥1 answer or where there were missing values. Abbreviations: BMI, body mass index.

**Table 2:** Medical history of women with polycystic ovary syndrome.

Variable	Value ^a^
**Has a PCOS diagnosis from a health practitioner**	98.5 (1150)
By a general practitioner	54.5 (636)
By a gynecologist	20.7 (241)
By an endocrinologist	18.6 (217)
By other	5.1 (60)
**Prescribed medication to regulate menstrual cycle or promote ovulation**	62.0 (724)
Metformin	37.6 (439)
Oral Contraceptives	30.7 (358)
Clomid or Letrozole	7.0 (82)
Injectable insulin sensitizers	0.4 (5)
Other	11.8 (138)
**Currently have excess body hair**	80.7 (942)
**Currently has acne**	56.2 (656)
**Current periods**	
Regular (1/month)	33.8 (394)
Irregular (>1/month)	38.0 (443)
Irregular (<1/month)	9.9 (115)
Absent (none within last 6 months)	18.2 (212)
**Presence of comorbidity**	
Infertility	38.2 (446)
Hypercholesterolemia	15.0 (175)
Hypertension	15.0 (175)
Hypothyroidism	13.3 (155)
Gastro-esophageal reflux disease	12.7 (148)
Irritable bowel syndrome with GERD	10.3 (120)
Hypertriglyceridemia	10.1 (118)
Eating Disorder	8.5 (99)
Steatosis	7.5 (88)
Irritable bowel syndrome without GERD	5.5 (64)
Type 2 Diabetes	5.0 (58)
Other	19.1 (223)

^a^ Data were reported using % (N). Percentages are expressed as a proportion of the total study sample (n = 1167). Percentages do not add up to 100% where survey respondents could provide ≥1 answer or where there were missing values. Abbreviations: GERD, Gastro-esophageal reflux disease.

**Table 3:** Knowledge of formal dietary and physical activity guidelines and special diets and physical activity plans followed by women with polycystic ovary syndrome.

Variable	Value ^a^
**Aware of national or international government guidelines for a healthy lifestyle in PCOS**	6.4 (75)
**Currently follows formal dietary guidelines to manage PCOS**	33.3 (389)
American Diabetes Association	23.9 (93)
MyPlate	13.4 (52)
Dietary Guidelines for Americans	10.5 (41)
DASH	10.0 (39)
American Heart Association	7.7 (30)
American Cancer Association Society	0.8 (3)
Other ^b^	58.4 (227)
Low energy/low calories (includes Weight Watchers and other	18.9 (43)
popular diet industry programs)	
Combination of diets (e.g., a plant-based ketogenic diet)	14.5 (33)
Paleo	9.7 (22)
Low-glycemic	8.8 (20)
Low-carbohydrate/high-protein	8.4 (19)
PCOS specific plans (includes	6.6 (15)
Specialized (includes IBS friendly, autoimmune, or GAPS)	5.3 (12)
Ketogenic	4.4 (10)
Own research (though specific diet not defined)	4.0 (9)
Whole food diet	2.6 (6)
Low carb only	2.2 (5)
Mediterranean	0.9 (2)
Vegetarian	0.9 (2)
Bariatric surgery	0.9 (2)
Dietary principles outlined by NHS and/or WHO	0.9 (2)
Sugar free	0.4 (1)
Did not provide a written response	10.6 (24)
**Currently follows a special diet to manage PCOS**	57.3 (667)
Low-carbohydrate/high-protein	63.4 (423)
Low-glycemic	36.6 (244)
Gluten-free	31.8 (212)
Dairy-free	30.4 (203)
Low-energy/calorie	19.8 (132)
Soy-free	17.5 (117)
Paleolithic/Paleo	13.5 (90)
Low-fat	12.7 (85)
Vegetarian/vegan	12.0 (80)
High omega-3	9.3 (62)
Ketogenic	4.6 (31)
Low-FODMAP	2.7 (18)
**Aware of 2008 Physical Activity Guidelines for Americans**	13.4 (156)
**Currently follows formal PA guidelines to manage PCOS**	16.2 (189)
**Engages in regular physical activity ^c^**	52.4 (611)
Combination of strength and cardio training (including HIIT)	58.8 (359)
Combination of cardio (including walking and swimming, walking	16.4 (100)
and cycling, running and cycling, etc.)	
Walking only	13.9 (85)
Yoga/Pilates	3.3 (20)
Strength training only	2.3 (14)
Running only	1.1 (7)
Cycling only	1.1 (7)
Aqua aerobics/swimming only	0.5 (3)
Horse riding	0.3 (2)
Manual work (including gardening and cleaning)	0.3 (2)
Organized sport (including basketball and football)	0.3 (2)
Did not provide a written response	1.6 (10)

^a^ Data were reported using % (N). Bolded percentages are expressed as a proportion of the total study sample (n = 1167). All other percentages are expressed as a proportion of their corresponding category (e.g., of the 33.3% of women who report following formal guidelines, 10.5% used the Dietary Guidelines for Americans). Percentages do not add up to 100% where survey respondents could provide ≥1 answer or where there were missing values. ^b^ Open-ended answers were coded using content analysis and are presented numerically as a proportion of the ‘other’ category (e.g., 18.9% of women who selected ‘other’ when asked whether they follow any formal nutrition guidelines were following a low energy/calorie diet). ^c^ Open-ended answers were coded using content analysis and are presented numerically as a proportion of respondents who reported that they ‘engaged in regular physical activity’. Abbreviations: DASH, Dietary Approaches to Stop Hypertension; GAPS, Gut and Psychology Syndrome, NHS, National Health Service; WHO, World Health Organization

**Table 4:** Self-management strategies used among women with PCOS.

**Diet**	**Overall Dietary Management Score**		2.99 ± 1.22
**Cognitive strategies**	Mean ± SD	Behavioral strategies	Mean ± SD
I make plans to change my diet/drinking habits.	3.39 ± 1.10	I read labels to help me choose healthy foods	3.50 ± 1.23
I look for information about nutrition and healthy eating from books, magazines, internet etc.	3.30 ± 1.31	I eat healthy food	3.24 ± 0.91
If I don’t eat healthy foods, I think about ways to do better next time.	3.21 ± 1.19	I watch what I eat	3.19 ± 1.13
I can stop myself from over eating	2.97 ± 1.17	I watch my weight	3.23 ± 1.25
I make sure I have time to prepare healthy meals	2.84 ± 1.17	I have food available for quick healthy meals	2.87 ± 1.15
I decide what to eat at the last minute	2.79 ± 1.01	I replace snacks with healthy alternatives	2.98 ± 1.00
I say positive things to myself about eating healthy food	2.79 ± 1.28	I weigh myself regularly	2.94 ± 1.37
I seek information about my weight from my GP	2.09 ± 1.19	I keep track of what I eat and how much I should eat	2.62 ± 1.23
**Physical activity**	**Overall physical activity management score**		2.96 ± 1.27
**Cognitive strategies**	Mean ± SD	Behavioral strategies	Mean ± SD
I know when I should do more activity	3.64 ± 1.14	I do things to make walking or other activities enjoyable	2.90 ± 1.19
I think about the benefits of being active	3.64 ± 1.10	I plan ahead of time to be active	2.78 ± 1.25
I try to think more about the benefits of physical activity and less the hassles of being active	3.28 ± 1.11	I can stick to my plans and be active each week	2.68 ± 1.09
When I set goals I choose activities that I enjoy	3.17 ± 1.12	I keep track of how much physical activity I do each week	3.39 ± 1.24
I read articles about the benefits of being active from magazines, books or the internet.	3.06 ± 1.20	When I get off track with my physical activity I find ways to get back on track	2.54 ± 1.23
		I ask my friends and family to walk with me to help me stay active	2.40 ± 1.30
		I make back up plans to make sure I get enough physical activity	2.10 ± 1.20

Abbreviations: GP, general practitioner; PCOS, polycystic ovary syndrome; SD, standard deviation.

## Data Availability

The data presented in this study are available upon request from the corresponding author.

## Appendix A—Questions Included in Survey Instrument Relating to Knowledge and Sources of Diet and Physical Activity Information

Awareness of PCOS Lifestyle Guidelines

Are you aware of any specific national or international government guidelines for having a healthy lifestyle (diet and physical activity) for women with PCOS?

- Yes
- No

Knowledge of Formal Dietary Guidelines, Special Diets Followed and Sources of Diet Information

Do you currently follow any formal nutrition guidelines to manage your PCOS?

- Yes
- No

If yes, have you ever used any of the following nutritional guidelines to manage your PCOS?

- The Dietary Guidelines for Americans
- My Plate
- Dietary Approaches to Stop Hypertension (DASH)
- American Heart Association
- American Diabetes Association
- American Cancer Association
- Other

If you selected other, please specify below:

___________________________________

Do you currently follow a special type of diet?

- Yes
- No

If yes, please select from the list below:

- Low energy/calories
- Low fat
- Low carbohydrate/high protein
- Low glycemic/glycemic load
- Gluten free
- Dairy free
- Soy free
- Ketogenic
- Vegetarian/vegan
- Paleolithic/paleo diet
- High omega-3
- Low FODMAPs

Was this diet recommended to you by a health care professional?

- Yes
- No

If yes, who recommended this diet to you?

- Doctor
- Nurse
- Registered Dietitian
- Nutritionist
- Health Coach
- Naturopathic Doctor
- Other

If you selected other, please specify below:

___________________________________

If a health care professional did not recommend this diet to you, where did you get the nutrition information for this diet?

- Family
- Friends
- Websites
- Social Media
- Television
- Radio
- Other

If you selected other, please specify below:

___________________________________

Knowledge of Formal Physical Activity Guidelines and Sources of Physical Activity Information

Do you currently follow any published physical activity guidelines to manage your PCOS?

- Yes
- No

Are you aware of the 2008 Physical Activity Guidelines for Americans?

- Yes
- No

Do you currently exercise regularly?

- Yes
- No

If so, what does it involve?

___________________________________

Was this exercise plan recommended to you by a healthy professional?

- Yes
- No

If no, from whom or where did you get the information for this exercise plan from?

- Family
- Friends
- Websites
- Social Media
- Television
- Radio
- Other

If you selected other, please specify below:

___________________________________

If yes, who recommended this exercise plan to you?

- Doctor
- Nurse
- Registered Dietitian
- Nutritionist
- Health Coach
- Naturopathic doctor
- Other

If you selected other, please specify below:

___________________________________

## Appendix B

**Table A1:** Sources of information for diet and physical activity plans followed by women with polycystic ovary syndrome—findings from content coding of written responses.

Category ^a^	Value ^b^
**‘Other’ health professionals (not listed as pre-defined answers) that provided diet advice**	
Endocrinologist	5.8 (4)
Acupuncturist	2.9 (2)
Allergist	1.4 (1)
Fertility specialist	1.4 (1)
Functional medical doctor	1.4 (1)
Personal trainer	1.4 (1)
Holistic practitioner	1.4 (1)
Did not provide a written response	84.1 (58)
**‘Other’ health professionals (not listed as pre-defined answers) that provided physical activity advice**	
Exercise physiologist or physiotherapist	30.6 (15)
Personal trainer	24.5 (12)
Endocrinologist	8.2 (4)
Chiropractor or kinesiologist	6.1 (3)
PCOS forum	2.0 (1)
Did not provide a written response	71.4 (35)
**‘Other’ alternative sources (not listed as pre-defined answers) that provided diet advice**	
Books or magazines	20.0 (29)
Conducted own research	17.2 (25)
Personal trainer	3.4 (5)
Weight loss program	2.1 (3)
Combination (e.g., books, friends, and websites)	1.4 (2)
Others with PCOS	1.4 (2)
PCOS forum	0.7 (1)
Did not provide a written response	46.2 (67)
**‘Other’ alternative sources (not listed as pre-defined answers) that provided physical activity advice**	
Conducted own research	72.8 (155)
Personal trainer	12.7 (27)
Employment	3.3 (7)
Books or magazines	1.9 (4)
Government guidelines (e.g., NHS)	0.9 (2)
Wii fit console	0.5 (1)
Did not provide a written response	92.0 (196)

^a^ Categories were developed using conventional content analysis. ^b^ Data were reported using % (N). Percentages are expressed as a proportion of the ‘other’ category (e.g., of the 23.5% of women who selected ‘other’ when asked whether they sort dietary advice from a health professional, 5.8% received their advice from an endocrinologist).
